# Syringeal Specialization of Frequency Control during Song Production in the Bengalese Finch (*Lonchura striata domestica*)

**DOI:** 10.1371/journal.pone.0034135

**Published:** 2012-03-27

**Authors:** Kristen R. Secora, Jennifer R. Peterson, Catherine M. Urbano, Boah Chung, Kazuo Okanoya, Brenton G. Cooper

**Affiliations:** 1 Department of Psychology, Texas Christian University, Fort Worth, Texas, United States of America; 2 Department of Cognitive and Behavioral Sciences, The University of Tokyo, Tokyo, Japan; Claremont Colleges, United States of America

## Abstract

**Background:**

Singing in songbirds is a complex, learned behavior which shares many parallels with human speech. The avian vocal organ (syrinx) has two potential sound sources, and each sound generator is under unilateral, ipsilateral neural control. Different songbird species vary in their use of bilateral or unilateral phonation (lateralized sound production) and rapid switching between left and right sound generation (interhemispheric switching of motor control). Bengalese finches (*Lonchura striata domestica*) have received considerable attention, because they rapidly modify their song in response to manipulations of auditory feedback. However, how the left and right sides of the syrinx contribute to acoustic control of song has not been studied.

**Methodology:**

Three manipulations of lateralized syringeal control of sound production were conducted. First, unilateral syringeal muscular control was eliminated by resection of the left or right tracheosyringeal portion of the hypoglossal nerve, which provides neuromuscular innervation of the syrinx. Spectral and temporal features of song were compared before and after lateralized nerve injury. In a second experiment, either the left or right sound source was devoiced to confirm the role of each sound generator in the control of acoustic phonology. Third, air pressure was recorded before and after unilateral denervation to enable quantification of acoustic change within individual syllables following lateralized nerve resection.

**Significance:**

These experiments demonstrate that the left sound source produces louder, higher frequency, lower entropy sounds, and the right sound generator produces lower amplitude, lower frequency, higher entropy sounds. The bilateral division of labor is complex and the frequency specialization is the opposite pattern observed in most songbirds. Further, there is evidence for rapid interhemispheric switching during song production. Lateralized control of song production in Bengalese finches may enhance acoustic complexity of song and facilitate the rapid modification of sound production following manipulations of auditory feedback.

## Introduction

Lateralized behaviors are controlled preferentially by one side of the body. It was once thought that lateralization of function only occurred in humans, but it is now known that the control of complex, learned behaviors is specialized to one side of the body in diverse species including fish, parrots, crows, rodents and chimpanzees [1]–[4]. The functional significance of specializing task control to one side of the body over the other remains unknown. Language production in humans is an example of a lateralized behavior [5]. When compared to the right hemisphere, the cortical areas controlling language production and comprehension are larger and during speech production are more active on the left side of the brain [6], [7]. This lateralization is also observed in congenitally deaf children exposed to early sign language, suggesting that hemispheric specialization is tied to language and not just speech production [8].

Singing in songbirds is an example of a lateralized behavior which shares many similarities with human speech [9]. Songbirds produce complex songs that include two-voice sounds generated by a bipartite vocal organ (syrinx). The bipotential sound generation gives rise to a rich and diverse acoustic phonology. The specialization of song production provides a unique animal model system for exploring the functional significance of lateralized behaviors. Further, because both sides of the syrinx perform specific functions at specific times during the song, and each side of the brain innervates only the ipsilateral syringeal muscles, the role of rapid interhemispheric switching of motor control during song can also be studied [10]–[13].

Studies of lateralized control of song began with resection of the tracheosyringeal branch of the hypoglossal nerve, NXIIts. This nerve is a mixed cranial nerve, controlling the ipisilateral syringeal muscles. In Waterslager canaries (*Serinus canaria*), resection of the left NXIIts nerve eliminated 85% of the syllables, whereas removal of the right NXIIts nerve only affected 12% of the syllables [14], [15]. This particular specialization arose from domestication and artificial selection for song features because domestic canaries (*Serinus canaria domestica*), which have not been selectively bred, use the left and right syringeal sound generators equally to produce their song [16].

In most songbirds, the different syllables of the song are produced by one or both sides of the syrinx, but in all species the left and right sides of the syrinx make different contributions to frequency control. Recording of airflow through the left and right bronchus has revealed that in domestic canaries (*Serinus canaria*), zebra finches (*Taeniopygia guttata*), brown-headed cowbirds (*Molothrus ater*), northern cardinals (*Cardinalis cardinalis*), brown thrashers (*Toxostuma rufum*) and gray catbirds (*Dumetella carolinensis*), the right syringeal sound generator produces higher frequency sounds, while the left side of the syrinx generates lower frequency sounds [10], [16]–[23]. The sole exception reported thus far is juvenile Australian magpies (*Gymnorhina tibicen*); in this species the right sound source produces lower frequency notes, and the left side of the syrinx produces higher frequency components of their song [24]. Thus, within the vast number of songbird species (*circa* 4000 species), the left and right syringeal sound sources can be used in different ways to differentially control the spectral and temporal properties of the bird's song.

Although syringeal sound control is lateralized, respiratory song motor patterns are not. Even when birds are singing by rapidly alternating between notes generated on the left or right side of the syrinx, or producing sound unilaterally, the left and right abdominal respiratory muscles are activated equally [25]. Therefore, sound production requires coordinating a symmetrical respiratory motor pattern with an asymmetrical syringeal motor pattern. Respiratory air pressure is critical for song production to induce the oscillatory behavior of the medial and lateral labia within the syrinx [26]. Air pressure is as stereotyped as the acoustic structure of song syllables. When different zebra finches sing similar song syllables they generate comparable air pressure patterns [27]. In a suboscine, the Great Kiskadee (*Pitangus sulfuratus*), respiratory air pressure controls the fundamental frequency of sound, even in the absence of syringeal muscular control [28]. Respiratory pressure patterns can also be used to identify syllables when birds are devoiced by preventing syringeal gating of airflow [29]. In sum, respiratory muscle activity is a symmetrical motor pattern, can control acoustic phonology independent of syringeal muscular control, and can be used to identify motor gestures underlying song production when syringeal gating is compromised.

The Bengalese finch (*Lonchura striata domestica*) is a domesticated species that is closely related to the zebra finch. Bengalese finches have received considerable attention because they rapidly modify the acoustic structure of their song following deafening or experimental manipulations of auditory feedback [30]–[33]. Damage to the left premotor nucleus HVC (acronym used as proper name) causes greater change in the acoustic structure of song than similar damage to the right HVC [34]. Additionally, the acoustic structure of the Bengalese finch distance call changes more following left NXIIts nerve resection compared to denervation of the right side of the syrinx [35].

Despite these studies, a characterization of how the two sides of the Bengalese finch syrinx interact during the production of normal song has not been studied in detail. Nerve resection alone is problematic because passive sound production can occur when air pressure reaches phonatory threshold. Bronchial airflow measurements or unilateral plugging of the bronchus circumvent this problem, but these techniques are technically difficult in small birds and can interfere with normal sound production [16], [18]. To avoid these problems, we used a combination of syringeal denervation in a larger sample of Bengalese finches, followed by unilateral devocalization or air pressure recordings before and after unilateral nerve resection in a smaller sample of birds. Air pressure recordings enabled syllable identification prior to and following unilateral neurotomy, which allowed for the description of how acoustic structure at the level of the individual syllable changed as a result of nerve resection. The data from these experiments provide clear evidence for syringeal specialization of acoustic control in Bengalese finches and they also illustrate the high likelihood for rapid interhemispheric switching of motor control during Bengalese finch song production.

## Materials and Methods

### Ethics Statement

This study was conducted with approval from the Texas Christian University Institutional Animal Care and Use Committee (# 0805) in an animal facility inspected and approved by the United States Department of Agriculture. All surgical procedures were carried out under isoflurane anesthesia (1–2%), and all efforts were made to minimize pain and discomfort.

### Animals and Recording Procedures

Nineteen adult male Bengalese finches were used in this study. They were given seed and water ad libitum and vegetables mixed with vitamins every day. Animals were housed in communal cages in a room on a 14∶10 light∶dark cycle. While birds were undergoing experimental manipulations, they were housed individually in small cages (31.8×10.5×25.4 cm) contained in a sound-attenuating box (78.7×33×33 cm). All four sides of the sound-attenuating box were lined with 1″ thick acoustic foam (Auralex Acoustics, Indianapolis, IN). A microphone was suspended 14 cm above the perch in the center of the cage.

### Surgical procedures

#### Tracheosyringeal nerve resection

Intact song was recorded in eleven birds, and then they were anesthetized with isoflurane (1–2%) and feathers removed from the ventral surface of the neck. An incision was made in the skin over the middle portion of the trachea. Either the right (n = 5) or the left (n = 6) NXIIts nerve was dissected free from the connective tissue, and a 4–5 mm section removed to discourage regeneration. The incision was sutured and the bird was monitored until it perched. Birds usually recovered and resumed singing within the first several days following nerve resection. Song was recorded continuously until two to three days of song was recorded. These initial songs provided the samples for the post-surgery recordings.

#### Unilateral devocalization

Four birds were anaesthetized with Chloropent (4 µL/g, Intramuscular injection). The bird's thoracic cavity was opened and a small incision was made in the interclavicular air sac to expose the syrinx. Either the right (n = 2) or left (n = 2) 3^rd^ bronchial semi-ring was extracted with a fine pair of forceps. The air sac and body were sealed using tissue adhesive and sutures. The birds were then returned to their cages to recover. Three of the four birds sang within three days following surgery (1 left and 2 right devocalized birds). One bird did not sing for the first week following left devocalization, and his song was not recorded again until 30 days post-surgery. After song was recorded for two days, birds were sacrificed and extraction of the cartilage and sealing of the bronchial lumen was verified by two individual raters who were not aware of which side was devocalized. There was 100% agreement between raters. In all cases reported here, the bronchial lumen sealed itself with tissue growth between the medial and lateral wall. This functioned to effectively plug the bronchus and prevent airflow through that side of the syrinx.

#### Air pressure and tracheosyringeal nerve resection

Air pressure was recorded in four birds before and after nerve resection. Birds were anesthetized using isoflurane (1–2%), and a small cannula (Silastic tubing, 0.76 mm I.D., Dow Corning, Midland, MI) was inserted below the rib cage into a thoracic air sac and held in place with suture looping around the 2^nd^ and 3^rd^ ribs [27], [29]. The suture was tied to cannula and a small amount of tissue adhesive was added to seal the body wall to the air sac. The free end of the cannula was connected to a pressure transducer (Fujikura FPM 02PG; Santa Clara, CA), amplified 100×, low pass filtered (6 kHz cutoff, Brownlee Precision, Model 440, Santa Clara, CA) and song was recorded simultaneously with respiration. After birds sang for one to three days, the left (n = 2) or right (n = 2) NXIIts nerve was removed following the procedures described above for the acoustic analyses. Then, song and air pressure were recorded following nerve resection for two to four days. Because the general pattern of air pressure is retained in the absence of syringeal control [29], respiratory motor patterns were used to confirm syllable identity prior to, and following nerve damage. Syllable identity between pre-operative and post-operative recordings was determined by visual inspection of the air pressure patterns. Two raters independently identified the syllables, and inter-rater agreement was calculated.

### Experimental procedures

Baseline song was recorded from each bird for two to five days prior to undergoing surgical procedures. The acoustic data were amplified and high pass filtered (300 Hz) prior to digitization (Brownlee Precision Amplifiers, Model 440, San Jose, CA). Data were recorded onto a computer using an analog to digital converter (National Instruments, NI USB-6251, Austin, TX) and Avisoft Recorder software (Avisoft Bioacoustics, Berlin, Germany). Data were saved to disk when the sound amplitude exceeded a user defined amplitude threshold. Each file was recorded as a time stamped 16 bit .wav file directly onto the hard drive. In order to avoid missing song, particularly in the post-surgery recordings, the trigger was set to a conservative low threshold. In some cases the preamplifier gain had to be increased to capture the song following left nerve resection; because the absolute change in the amplifier gain was not recorded, sound amplitude comparisons between experimental conditions were not possible in this part of the study. However, when song and air pressure were recorded together, the amplitude of the air pressure pattern was used to trigger song recordings. For these recordings, we were able to compare relative sound amplitude levels prior to and following nerve resection because the amplification of the microphone signals did not have to be adjusted to identify onset of song.

### Data analysis

#### Average song feature analysis

Average spectral and temporal features of songs were measured and compared before and after nerve resection. A subset of 75 songs from morning, afternoon and evening recordings in the pre- and post-surgery recordings were used for the analysis. No differences between recordings at different times of the day were observed. Songs were then digitally filtered using a Finite Impulse Response bandpass filter (high cut 0.3 kHz; low pass 9 kHz) prior to acoustic analysis. The syllables within the song (including introductory notes at the beginning of the song) were segmented based on a user defined amplitude threshold. Syllables onset and offset were determined by this threshold, which demarcated continuous acoustic events on a spectrogram.

The following acoustic features of syllables were measured, fundamental frequency (f_0_), peak frequency, duration, and interval (SASLab Pro, Avisoft Bioacoustics, Berlin, Germany). Complementary analyses of acoustic features of the syllables, frequency modulation (FM), amplitude modulation and Wiener entropy (entropy) were calculated using Sound Analysis Pro, courtesy of O. Tchernichovski [36]. SASLab and Sound Analysis Pro were both used because they are the standard analysis programs used in the birdsong field; using both programs will enable more direct comparisons to previous and future studies in this species. Additionally, these programs provide complementary descriptions of the spectral changes in song structure following neurotomy.

To compute the f_0_ of individual syllables only the syllables with clear harmonic stacks were analyzed. The f_0_ was determined visually from the mean power spectrum generated from five repetitions of the syllable and was defined as the lowest integer multiple of the energy peaks within the mean spectrum. The peak frequency was the frequency with the greatest acoustic energy within a syllable. Duration was defined as the length of time from the onset to the offset of the syllable. Interval was the time between syllables (offset of one syllable to the onset of the next). In the automatic measurements of SASLab, the value given as integer includes the syllable duration (extends from the offset of one syllable to the offset of the next). The preceding syllable duration was subtracted from the interval measurement to determine the intersyllable interval. Sound Analysis Pro was also used for analysis of frequency modulation (FM), amplitude modulation and entropy. FM is the mean slope of the changing frequencies during the segmented syllable. Entropy is a measure of the randomness of the signal in the frequency domain. The average feature for each parameter for all of the syllables sung within the bird's song were calculated and used as the datum for the pre- and post-surgery scores in the statistical analysis.

### Statistical Analyses

#### Average song feature analysis

A paired t-test was used to determine how the overall acoustic structure of the song changed following nerve resection. A mean score of the all of the syllables in each bird's song was calculated for each spectral and temporal parameter measured in SASLab (f_0_, peak frequency, duration, and interval), and Sound Analysis Pro (FM, amplitude modulation and Weiner entropy). Each bird provided a single average datum point for each measured parameter. Left and right nerve resection data were analyzed separately. Because of the small sample size in the devocalization experiments only descriptive statistical analyses of average song parameters were presented for this experiment.

#### Individual syllable analysis

**Figure 1:**
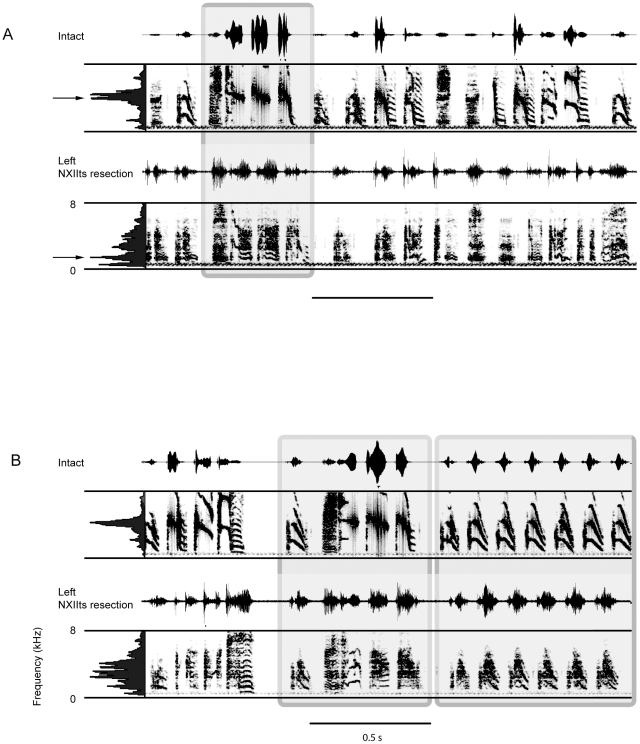
The left side of the syrinx controls the spectral features of song in the Bengalese finch. A) Spectrograms of the most severely changed song in a Bengalese finch before and after left syringeal denervation. In the intact song, the peak acoustic energy is centered at 4 kHz (indicated by the arrow to the left of mean spectrum), which is common in Bengalese finch songs. Following left syringeal denervation there was a remarkable loss of spectral control of the syllables and a decline in the peak frequency (arrow to the left of the mean spectrum). B) Another example of the effects of left syringeal denervation on the acoustic structure of the Bengalese finch song. In this individual, most of the syllables showed pronounced increase in the “noise”, which corresponds to an increase in entropy, and there was greater energy in the lower frequencies of the song (<4 kHz). Boxes around syllables indicate putative visually matched syllables to illustrate the difficulty in identifying syllables based solely on acoustic structure following left neurotomy.

Due to the drastic changes in syllable acoustic structure following left nerve resection (e.g., Figure 1), matching intact to post-operative syllables was not possible from the acoustic recordings without assuming syllable identity based on syllable order. Because of the complex and variable syntax of the Bengalese finch song, this assumption may not always be valid [37]. Further, because the average song feature analysis included all syllables sung by the bird, the average values for different spectral and temporal features could be biased by birds singing one syllable type more frequently than another. Therefore, air pressure recordings were used to identify syllables prior to and following the nerve resection, because respiratory patterns are minimally changed by NXIIts nerve damage. Matched syllables in the intact and post-operative recordings were identified by visual inspection of air sac pressure patterns by two independent raters. Ambient air pressure was estimated in the recordings by the midpoint between expiration and inspiration during a 2 to 5 second period of quiet respiration, and then song syllables were demarcated by continuous supra-atmospheric air pressurization. By this definition a syllable is a respiratory unit of motor production rather than an acoustic unit, however the two are usually identical [27], [38]. Within this positive pressurization, two acoustic features of the syllable were calculated, peak frequency (frequency with the highest energy content within the syllable) and sound amplitude (microphone Root Mean Square, RMS). These features were calculated for all of the syllables that were sung within the bird's song both before and after nerve resection. In these recordings, each of the four birds contributed 10–14 syllables for the analysis. Fifteen repetitions of these syllables before and after neurotomy were used to calculate an average syllable peak frequency and amplitude. A paired t-test was used to determine if the syllable peak frequency and RMS amplitude changed significantly following nerve resection. Left and right nerve resection data were analyzed separately.

Linear regression analyses were calculated to determine whether syllable peak frequency in the intact song was a significant predictor of the change in peak frequency induced by neurotomy. The intact peak frequency was used as the criterion variable and the predictor variable was the change in syllable peak frequency (ΔPF) induced by neurotomy. The ΔPF was the difference between post-neurotomy syllable Peak Frequency and the intact Peak Frequency (ΔPF = Peak Frequency_post_−Peak Frequency_intact_).Separate linear regression analyses were calculated for the left and right nerve resection data. Similarly, separate regression analyses were calculated to investigate how neurotomy affected sound amplitude. For the amplitude analysis, the intact syllable RMS was the predictor variable and the change in sound amplitude (ΔRMS) was the criterion variable. ΔRMS was equal to the change in microphone amplitude following sugery which was the difference between the post-surgery microphone amplitude and the intact amplitude (ΔRMS = RMS_post_−RMS_intact_).

## Results

### Left Syringeal Muscular Control is Critical for the Production of Higher Frequency Components of the Bengalese Finch Song

The most pronounced differences in the spectral features of the Bengalese finch song were observed following resection of the left NXIIts nerve, whereas right side nerve resection resulted in more modest changes in spectral features of song syllables. Figure 1 displays the effects of left nerve resection on acoustic structure of the song syllables for two different birds. Syllables became “noisier” which resulted in significant increase in Weiner entropy following left ts nerve resection, *t*_(5)_ = −21.3, *p*<.001. The amplitude of the song usually decreased substantially, and the microphone preamplifier gain had to be increased in several cases to trigger sound recordings. In the typical Bengalese finch song, the dominant acoustic energy is usually centered at 4 kHz. Following the left NXIIts nerve resection, almost all of the song syllables were spectrally degraded, but this appeared more striking for those syllables with harmonic notes with a higher peak frequency. Indeed, peak frequency declined significantly as a result of left NXIIts nerve resection, *t*(5) = 2.89, *p*<.05. Left NXIIts nerve resection resulted in significant changes in the average song features of peak frequency, , and entropy (Table 1).

**Table 1:** Changes in acoustic features following syringeal denervation.

	Left ts Resection		Right ts Resection	
Parameters	Pre-L	Post-L	Pre-R	Post-R
**Peak Freq (kHz)**	3.2+/−0.06	2.2+/−0.1**	2.8+/−1.3	2.7+/−1.2
**Duration (s)**	0.06+/−0.001	0.055+/−0.002	0.062+/−0.03	0.054+/−0.02**
**FM**	42+/−0.1	42+/−0.1	41+/−0.2	43+/−0.2
**Entropy**	−2.9+/−0.01	−1.6+/−0.005***	−2.9+/−0.3	−2.7+/−0.1

Values are means ± SEM. Effects of neurotomy were statistically compared with paired sample t-tests. *** *p*<0.001; ** *p*<0.01; * *p*<0.05.

**Figure 2:**
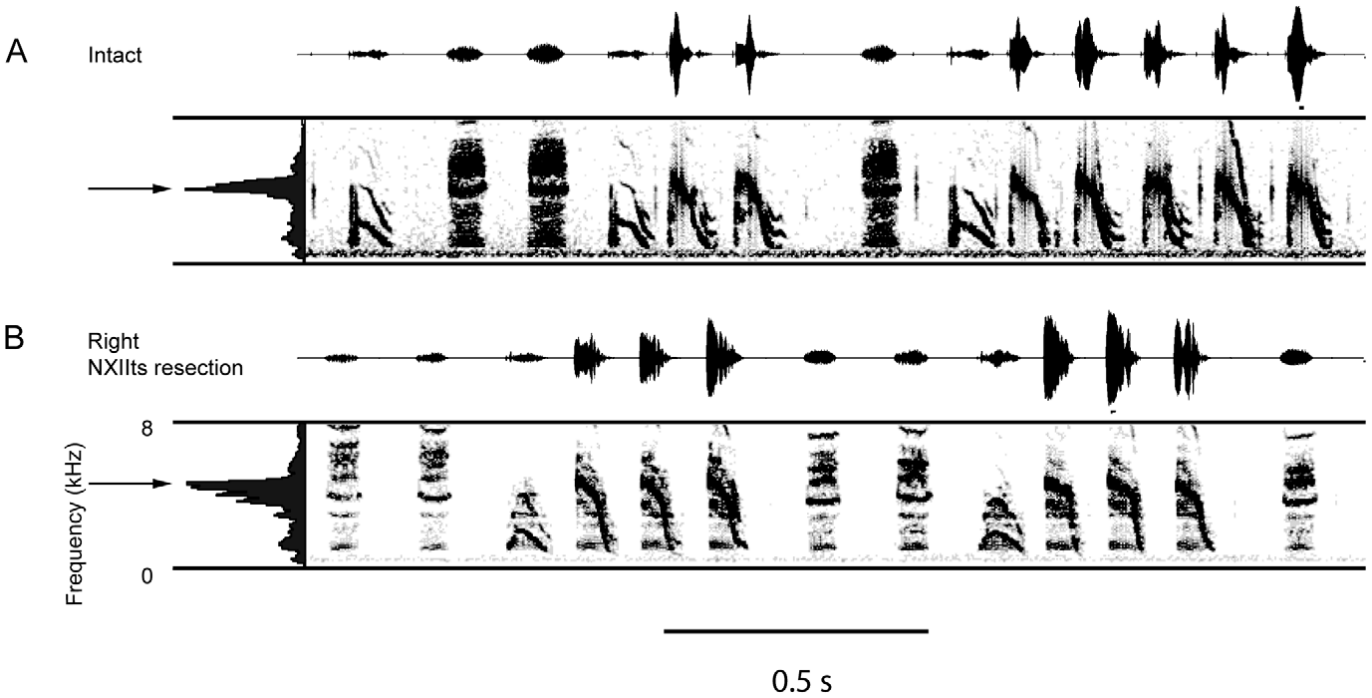
Denervation of the right syringeal muscles has little effect on the spectral features of song. Spectrograms of Bengalese finch song before and after right tracheosyringeal nerve resection. The spectral features of the song are very similar following right nerve resection. Arrows to the left of to the mean spectrum illustrate that the peak energy content remains centered around 4 kHz following right syringeal denervation, which is in contrast to the effects of left NXIIts nerve resection.

Figure 2 displays the pattern of data observed following right nerve resection in the song of a representative bird from this experimental group. Although some of the syllables were “noisier” following right syringeal denervation, the syllables with a higher peak frequency were retained, and the spectral energy remained centered around 4 kHz. Loss of neuromuscular control of the right side of the syrinx did not significantly change any of the spectral features of the song syllables (Table 1). The only statistically significant change induced by resection of the right NXIIts nerve was a significant decrease in syllable duration *t*(4) = 3.43, *p* = .013.

### Spectral Changes in Song Following Unilateral Devocalization Confirm Left Side Dominance for Higher Frequency Sounds

Denervation of the syrinx does not eliminate sound production from one side of the syrinx, rather it removes active muscular control of the movement and tension on the labia. This leaves the labia free to vibrate if air pressure conditions are sufficient to reach phonatory threshold. To eliminate passive sound production, we used unilateral devocalization to prevent sound production from one syringeal sound generator. Therefore, the sounds produced following unilateral devocalization were the result of the intact side only.

**Figure 3:**
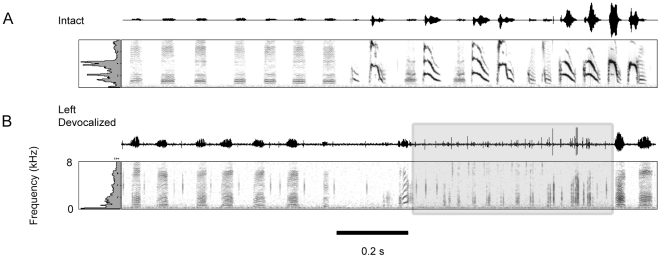
Left syringeal devocalization abolishes the majority of the Bengalese finch song. A) The intact song of a Bengalese finch is displayed in the top of the figure. The introductory notes are lower frequency notes, followed by a series of higher frequency song syllables. B) The introductory notes remained intact following left devocalization, but the remainder of the song syllables were no longer produced. The shaded box illustrates the presence of broadband noise “clicks” that appeared to coincide with the onset of the higher frequency song syllables. These “clicks” were repeatedly produced after the introductory notes, suggesting the syllable onsets of higher frequency syllables were produced by the right sound source, but the higher frequency notes were not produced without the left sound source.

Based on the nerve resection data, we predicted that devocalization of the left syringeal sound source would remove most of the higher frequency notes within the Bengalese finch song. The results from the left devocalization data confirmed this prediction. Figure 3 displays the severe change in song observed following left devocalization. The intact song contained introductory notes, followed by multiple syllable types with a varying peak frequency range. After the left syringeal sound source was removed, and therefore the bird sang with only the right sound generator, the bird produced lower frequency introductory notes, and then a series of broadband noise clicks (shaded box, Figure 3B). These clicks appear to coincide with the acoustic onset of the bird's song syllables, but the bird was unable to generate any of the higher frequency notes during song. Supplementary online material includes audio files of example song bouts recorded prior to (Audio S1), and following (Audio S2) left devocalization.

**Figure 4:**
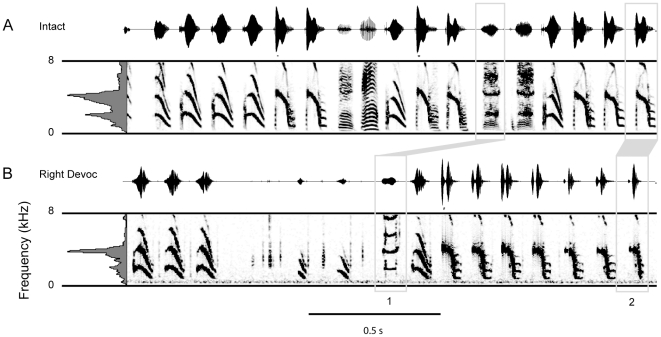
Right syringeal devocalization minimally changes the Bengalese finch song. A) An example of the intact song of a Bengalese finch, displaying a variety of the syllables sung by the bird. Following right syringeal devocalization the song remained very similar to the intact song, except the broadband noise that was produced during some of the syllables (Box 1) and at the onset of syllables (Box 2) was removed by right devocalization. The broadband noise is similar to the “clicks” that were produced by the left devocalized bird (Figure 3). These data are consistent with the right syringeal denervation showing a minimal change in spectral control of song. They further demonstrate that the decrease in syllable duration is due to a loss of sound at the onset of syllables.

To further confirm these findings, we devocalized the right sound source in two other birds. Data are shown for one bird, but the results were very similar in both birds. The right devocalization yielded subtle change in the overall song structure. Audio files of example song bouts recorded prior to devocalization of the right sound source (Audio S3) and following devocalization (Audio S4) are available in supplementary online material. The higher frequency acoustic energy was retained in all of the song syllables. However, lower frequency, higher entropy sounds were removed following right devocalization (Figure 4). Lower frequency, higher entropy sounds typically occur at the beginning of syllables and sometimes occur during a syllable. Bilateral syllable production is illustrated by the removal of lower frequency sound during the production of the syllable indicated in Box 1, Figure 4.

**Figure 5:**
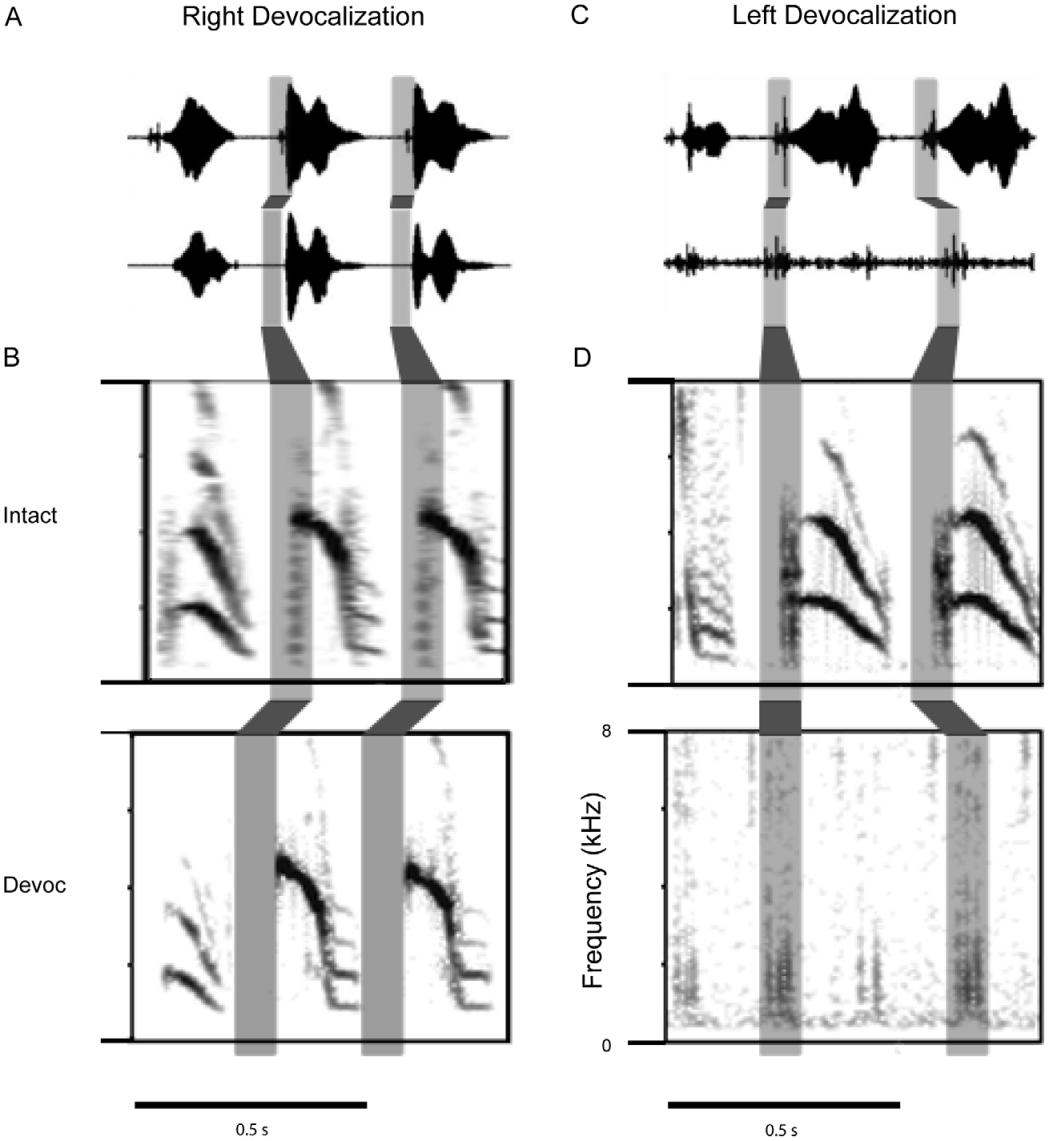
Model for interhemispheric switching during song production. A,B) An example of the microphone recording of higher peak frequency syllables in the intact recording and following right devocalization. The shaded area indicates the loss of sound at the syllable onset following right devocalization. The spectrograms of the intact and right devocalized syllables are shown with the sound at the syllable onset shaded in gray. This sound is eliminated by right devocalization. C,D) An example of the microphone recording in the intact syllables with a higher peak frequency before and the putative syllable onsets after left devocalization. The spectrograms are displayed below the microphone trace (C). The “clicks” likely represent the syllable onsets without sound production during the remainder of the higher frequency components of the syllables. These data illustrate the high likelihood for rapid interhemispheric switching during song production in Bengalese finches. Many syllables are produced by the syrinx with a lower frequency right side generated sound, followed by a rapid switch the left side for the higher frequency sound production.

In addition to bilateral sound production, many syllables have a unique pattern of right followed by left syringeal control of sound production. Figure 5 displays evidence that the right sound source is active at the beginning of higher frequency syllables, and then birds switch to left side sound production to generate the higher frequency and frequency-modulated notes within the syllable. Unilateral devocalization of the right sound source removed broadband noise at the onset of syllables, whereas left devocalization left only broadband noise at the putative onset of these syllables (Figure 3, Figure 5). Birds singing without the right sound source continued to sing higher frequency syllables, but these syllables were not produced with the broadband noise that occurred at the beginning of the syllable. The loss of the sound at the beginning of the syllable explains why right NXIIts resection decreased syllable duration. Therefore, the devocalization data confirm the lateralized frequency control, and also provide suggestive evidence for hemispheric switching during song production. The higher peak frequency syllables are generated by an initial right side contribution of lower frequency sound at the onset of the syllable, followed by a switch to the left sound source to produce higher frequency components of the syllable (Figure 5).

**Figure 6:**
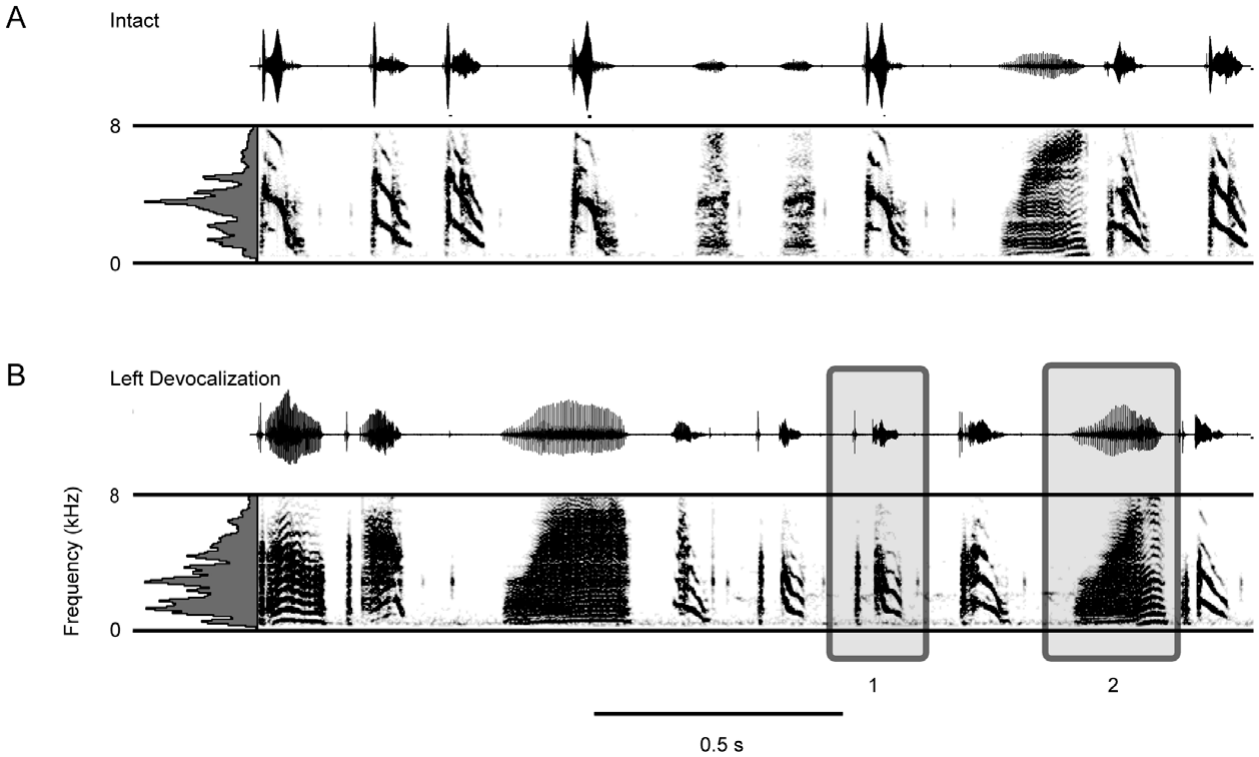
Left syringeal devocalization prevents sound production of syllables with a higher peak frequency. A) A representative example of the intact song from a Bengalese finch. B) Thirty days after devocalization the bird sang many of the song syllables from the intact song, with the important exception of syllables with a higher peak frequency. Box 1 indicates a silent area during the syllable that may correspond to the failure to produce higher frequency sounds without the left sound source. Box 2 shows that the lower frequency sounds are retained, and have pulse-tone like characteristics.

Because one devocalized bird did not sing immediately following surgery, we were able to assess song production after a month of recovery from surgery. In the intact song, the bird sang a mix of lower and higher frequency syllables (Figure 6A). Acoustic data were not recorded during the 30 day interval, but the song observed after 30 days showed that the bird retained or recovered many song syllables. The important exception was higher frequency notes were missing from the song syllables. We inspected all of the song syllables from both left devocalized birds, and computed power spectra (Hamming window, bandwidth 9.9 Hz, resolution 5.4 Hz) of well defined harmonic stacks in these birds. No sounds were generated with a f_o_ above 2.2 kHz following left devocalization. In fact, there were silent periods within syllables following left devocalization. These silent intervals appeared to correspond to the time when higher frequency notes would have been produced within the syllable (Box 1, Figure 6). Therefore, the results are consistent with the interpretation that the right side of the syrinx contributes to lower frequency sound production, and the left sound source produces higher frequency sounds. The data from this individual bird suggests that frequencies above 2.2 kHz are typically not produced by the right syringeal sound source, and instead are left side generated.

The specialization for lower frequency sound production is also illustrated by the findings that pulse-tone like sounds can be generated by Bengalese finches, and they are retained following left devocalization (Box 2, Figure 6B). The unilateral production of pulse tone sounds is similar to findings in hooded crows (*Corvus Corone cornix*), European starlings (*Sturnus vulgaris*) and Australian Magpies [24], [39]. The retention of these syllables in the left devocalized birds is consistent with specialized lower frequency sound production by the right side of the syrinx in Bengalese finches.

**Figure 7:**
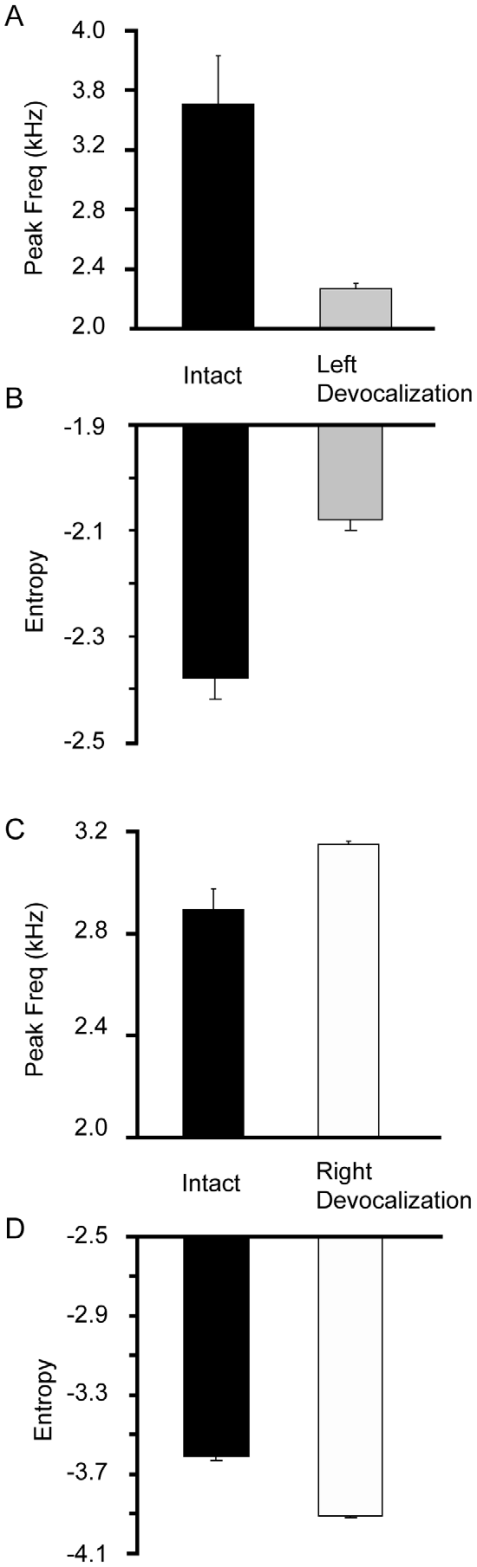
Descriptive analyses of the acoustic changes induced by left and right devocalization further illustrate the left/right frequency specialization in Bengalese finches. A & B) Removal of the left syringeal sound generator decreased the average peak frequency and increased the entropy in the songs recorded from these birds. C & D) In contrast, right syringeal devocalization increased average peak frequency and decreased the entropy in the songs recorded from these birds.

Descriptive statistical analyses of the syllables within the songs of the left devocalized birds showed that the removal of the left sound source caused the birds to sing with a lower average peak frequency but entropy increased (Figure 7A,B). In contrast, following right devocalization, peak frequency increased, whereas entropy decreased compared to the intact songs (Figure 7C,D). These data were consistent with the results from the larger sample of birds that underwent left or right neurotomy, showing that the left and right sound sources in Bengalese finches are specialized for higher and lower frequency sound production, respectively.

### Spectral and Amplitude Changes in Individual Syllables Following Neurotomy

**Figure 8:**
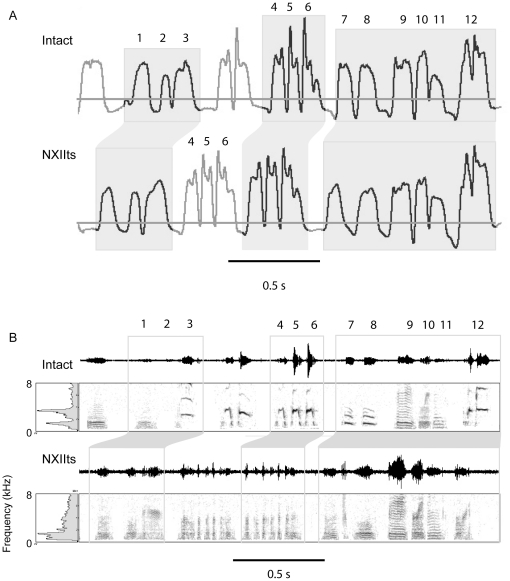
Air pressure was largely retained following denervation of the syrinx. A) Air pressure prior to nerve resection was used to identify syllables following nerve injury. The gray line indicates ambient pressure. Supra-atmospheric air pressure corresponds to expiration and sub-atmospheric air pressure to inspiration. The expiratory pulses during song provided a characteristic pattern, which was used to visually identify and match syllables after nerve resection. B) Acoustic structure cannot be used to match syllables following left syringeal denervation because it is dramatically changed following left NXIIts nerve resection.

Characterizing how individual syllables change following left nerve resection is problematic in acoustic recordings because of the pronounced acoustic deterioration that is commonly observed. To avoid this problem, air pressure was used to identify syllables pre and post-operatively because it was minimally changed following nerve resection even though acoustic structure was drastically altered (Figure 8). There was 100% agreement between the raters for the syllable identification between pre-operative and post-operative recordings. This experiment allowed for the analysis of how spectral and amplitude properties of individual syllables are affected by lateralized neurotomy. The intact and post-neurotomy peak frequency and sound amplitude (measured by RMS level) averages for each syllable were compared using a paired t-test. Following left nerve resection, the syllable peak frequency and sound amplitude decreased significantly, *t*_(21)_ = 7.73, *p*<0.001 and *t*_(21)_ = 3.0, *p*<0.01, respectively. In contrast, following denervation of the right side of the syrinx, neither peak frequency nor sound amplitude changed significantly for the individual syllables, *t*_(22)_ = −1.20, n.s., *t*_(22)_ = −1.53, n.s., respectively.

**Figure 9:**
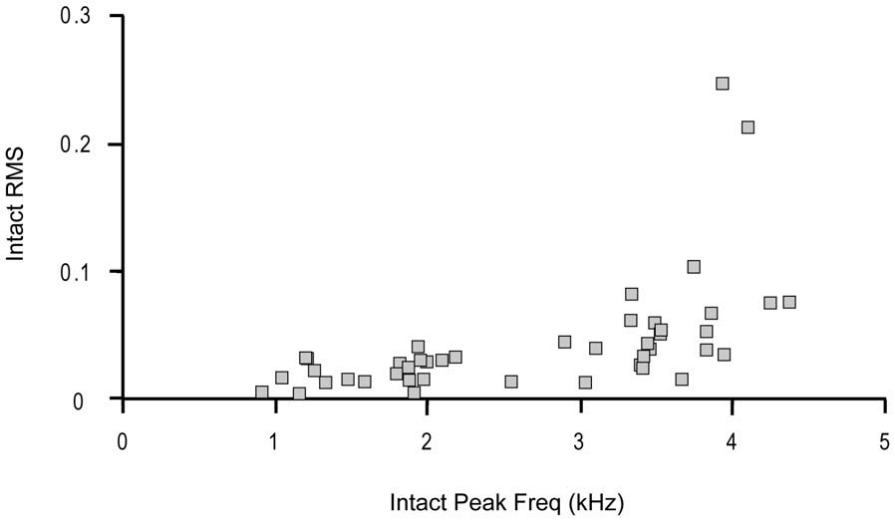
Frequency and amplitude of the Bengalese finch song syllables were linearly correlated. Each dot corresponds to an individual syllable. The higher frequency syllables were the louder syllables within the bird's song.

**Figure 10:**
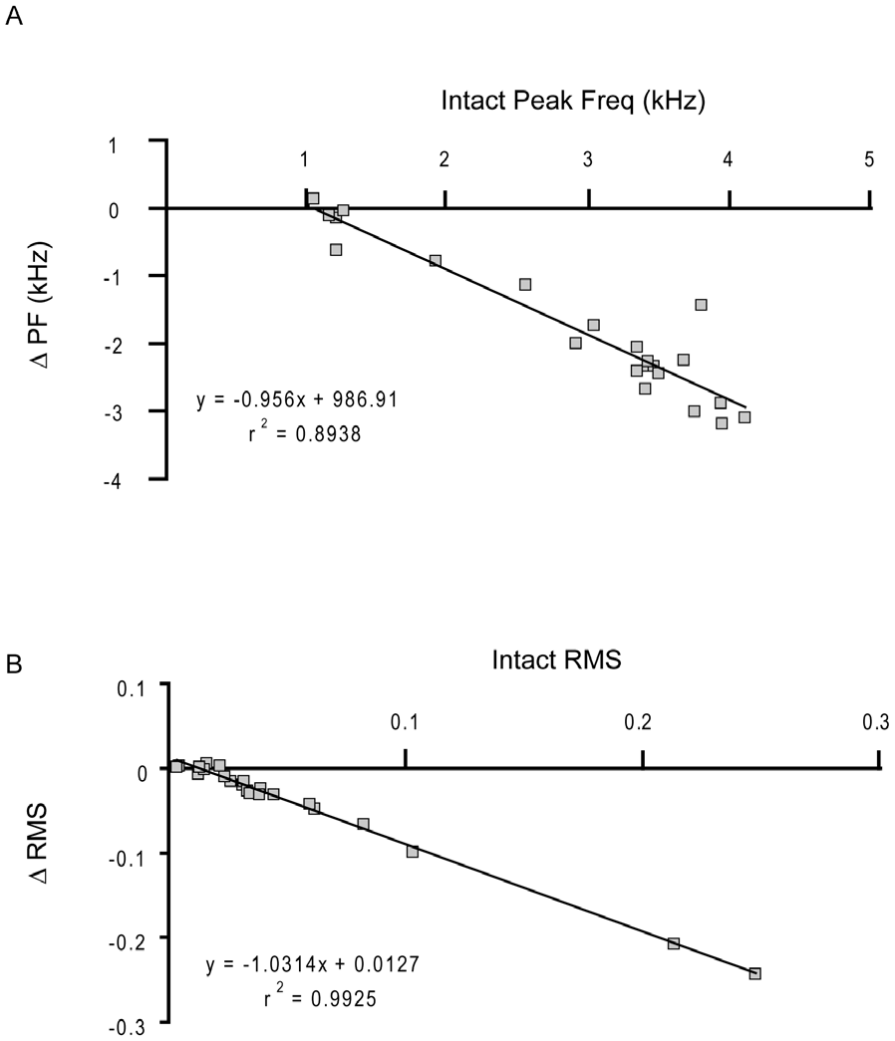
Intact peak frequency was a significant predictor of how much frequency will decrease following left syringeal denervation. A) Each dot corresponds to an individual syllable. Peak frequency was highly correlated with the change in peak frequency induced by left NXIIts nerve resection. The syllables with a peak frequency greater than 2 kHz decreased in frequency more than the syllables with a lower peak frequency (circa 1 kHz). B) Relative sound amplitude was also highly correlated with the decrease in sound amplitude induced by denervation of the left side of the syrinx.

In the intact air pressure recordings, the RMS sound amplitude and peak frequency for all of the syllables sung by the birds in this study were highly correlated, *r*_(43)_ = 0.63, *p*<0.001 (Figure 9). If louder, higher frequency syllables are controlled by the left side of the syrinx, then the greatest change in acoustic structure should be caused by loss of muscular control of the left sound source. Following left nerve resection, the intact peak frequency of a syllable significantly predicted the change in the peak frequency (ΔPF) following left NXIIts neurotomy, *β* = −0.956, *t*(20) = −12.98, *p*<0.001, *r* ^2^ = 0.89 (Figure 10A). Additionally, the intact sound amplitude also significantly predicted the change in sound amplitude (ΔRMS) following left neurotomy, *β* = −1.03, *t*(20) = −51.48, *p*<0.001, *r* ^2^ = 0.99 (Figure 10B). In Bengalese finch song syllables that have a higher peak frequency or sound amplitude, there was a highly linear decline in frequency and amplitude following left nerve resection. In contrast, those syllables with a lower peak frequency are the least likely to change in peak frequency or sound amplitude. Visual inspection of the scatterplots illustrates that syllables with a peak frequency greater than 2.0 kHz show the greatest decline in peak frequency. These data are consistent with the left devocalization data where birds were unable to produce syllables with a f_o_ greater than 2.2 kHz following left devocalization.

**Figure 11:**
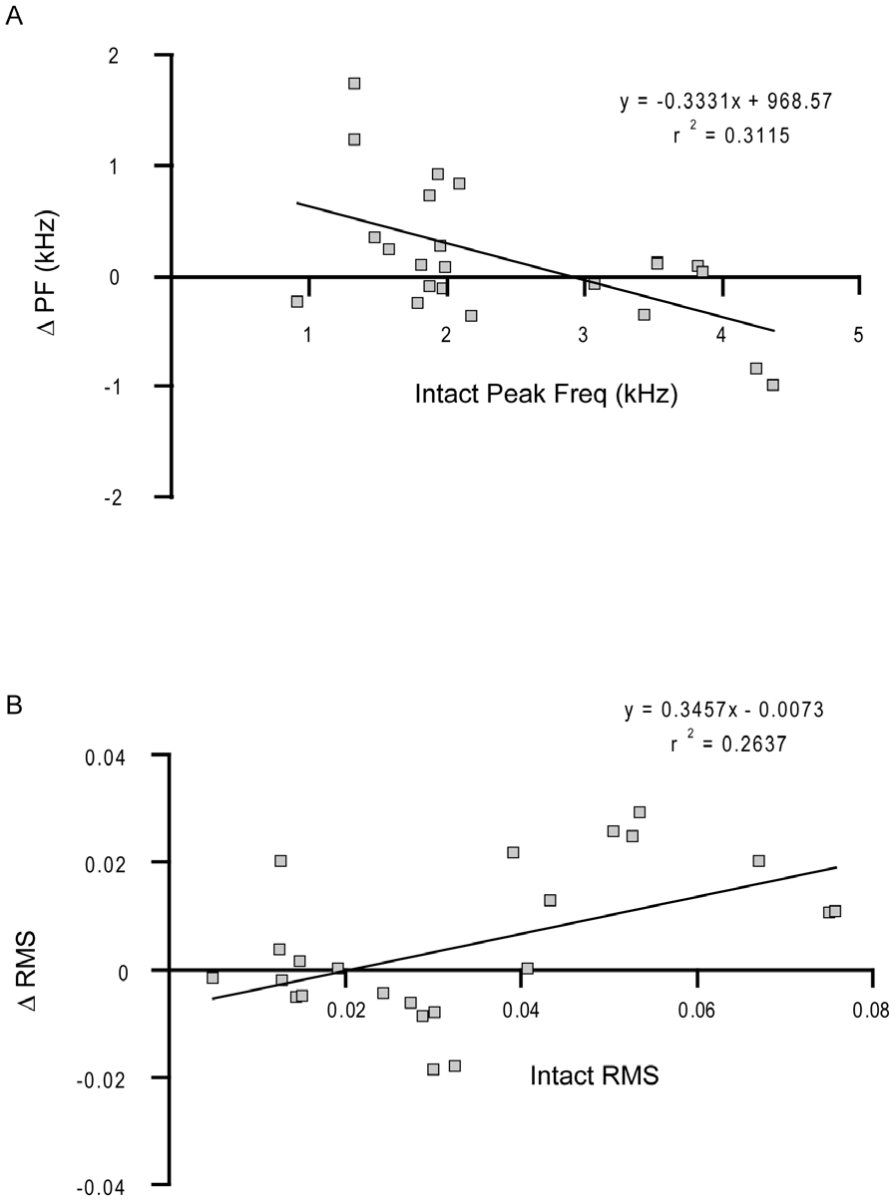
Denervation of the right side of the syrinx changes lower frequency syllables more than higher frequency syllables. A) Each dot corresponds to an individual syllable. The intact syllable peak frequency predicted the change in peak frequency following right NXIIts nerve resection; in contrast to denervation of the left side of the syrinx, lower frequency syllables (<2 kHz) were either unchanged or increased peak frequency following loss of right syringeal neuromuscular control. B) Further, right syringeal denervation caused the higher amplitude syllables to increase in sound amplitude.

Following right nerve resection, the intact peak frequency for each syllable was also a significant predictor of the ΔPF, *β* = −0.33, *t*(21) = −3.08, *p*<0.01, *r* ^2^ = 0.31, Figure 11A, however the relationship was not as strong as observed in the song syllables in the birds that underwent denervation of the left syringeal muscles. Intact sound amplitude was also a significant predictor of the *Δ*RMS following denervation of the right syringeal muscles, *β* = 0.35, *t*(21) = −2.74, *p*<0.02, *r* ^2^ = 0.26 (Figure 11B). This pattern is the opposite change in amplitude compared to left NXIIts neurotomy; right side nerve resection was more likely to increase the sound amplitude, particularly for higher amplitude song syllables.

## Discussion

These data provide strong evidence that Bengalese finches produce louder, higher frequency sounds with the left side of the syrinx, whereas the right side controls the softer, lower frequency syllables. This is the opposite pattern of lateralized frequency control observed in zebra finches, a closely related estrildine species. In Bengalese finches, the left side of the syrinx is the primary side in producing syllables of a higher peak frequency (>circa 2.0 kHz), but in zebra finches expiratory song syllables with a f_o_ above 3 kHz are produced exclusively by the right side of the syrinx [18]. Following left devocalization, no syllables were recorded with a fundamental frequency greater than 2.2 kHz. Syllables with the highest peak frequency were the most likely to decrease in peak frequency following left syringeal denervation. The opposite data pattern was observed following right syringeal denervation; the syllables with a peak frequency greater than 2 kHz were the least likely to change following denervation of the right side of the syrinx. Therefore, higher peak frequency syllables are generated by active muscular control of the left sound source, and these higher frequency syllables do not require active muscular control by the right side of the syrinx. Many of the Bengalese finch syllables contain broadband and lower frequency sounds, which were eliminated following devocalization of the right syringeal sound generator. Thus, there are separate roles for frequency production between the left and right sound generators in the Bengalese finch.

Why do Bengalese finches show such a strong lateralization of acoustic control? There are at least two possible reasons that are not mutually exclusive. First, there may be underlying morphological differences between the two sound generators in the Bengalese finch syrinx. A comparison of the acoustic structure of higher frequency sounds produced by the left side of the syrinx to the noisy structure of the right side generated sounds suggests that morphological variation in the sound generating labia could cause this acoustic specialization. A second possibility is that lateralized song in Bengalese finches arises due to neural constraints, rather than peripheral anatomical constraints. If the hemispheric specialization is due to neural constraints, then the lateralization of acoustic control could be due to either an auditory sensory or a motor production bias between the hemispheres. Evidence for lateralized auditory processing has been demonstrated in Mongolian gerbils (*Meriones unguiculatus*); in this species learning about frequency modulated sounds depends on the functional integrity of the right hemisphere [40]. Perhaps Bengalese finches also exhibit lateralized frequency processing. In Bengalese finches, the left hemisphere may be preferentially tuned to hearing higher frequency sounds and the right hemisphere could process lower frequencies of sound. Alternatively, lateralization could be driven by motor control of sound production in Bengalese finches. Future work can distinguish between these possibilities.

Okanoya has postulated that female sexual selection contributed to the origination of lateralized song production in Bengalese finches [37]. A comparison of the song of white-rumped munia (*Lonchura striata*) shows that they are more likely than Bengalese finches to sing syllables with broadband noise that appear to have higher entropy content [41]. The Bengalese finch is a domesticated strain of this species, and whether the white-rumped munias exhibit a similar pattern or different pattern of lateralization of acoustic production is currently unknown. Further studies of the white-rumped munia and Bengalese finches would be an excellent model system for exploring the role of intra- and inter-sexual selection in shaping acoustic features of song and the evolution of lateralized sound production [41].

### Lateralized Song Production Correlates with Song Plasticity

It is well established that Bengalese finches can modify the acoustic control of their song in response to real-time manipulations of auditory feedback [42], [43]. Bengalese finches can be trained to increase the fundamental frequency of a given syllable by playing white-noise when birds spontaneously sing lower frequency renditions of that syllable, and withholding the noise when birds spontaneously sing the same syllable with a higher frequency. This training causes birds to systematically shift the frequency of their vocalizations to avoid the noise playback during their song [42]. Earphones have also been placed on Bengalese finches to replay the bird's vocalizations with an upward or downward frequency shift (with a short temporal delay between vocalization and replay). When Bengalese finches heard themselves singing with a pitch-shift, they compensated for this auditory mismatch by reducing or increasing the fundamental frequency of their vocalizations [43]. In these studies, the higher frequency syllables were measured for their pitch-shift and for altering the fundamental frequency in response to white-noise training. These higher frequency syllables are generated primarily by only the left side of the syrinx (Figure 5). This is suggestive evidence that it may be easier for birds to modify acoustic features of song that are produced by only one side of the syrinx.

Additional evidence linking modification of learned song to lateralized sound production comes from the findings that higher frequency syllables are more likely to change following deafening than lower frequency syllables [44]. In contrast to Bengalese finches, zebra finches change their song more slowly following deafening. Interestingly, zebra finches produce most of the syllables of their song bilaterally; only a few higher frequency notes are produced by unilaterally [18]. Taken together, these data suggest a relationship between song plasticity and lateralized frequency control. The production of higher frequency syllables can be modified by training, and change rapidly following loss of auditory feedback. Perhaps learning to control one sound generator in isolation is easier and faster than having to modify the sound production of the left and right sound sources simultaneously. Thus, just as in other species where laterality is thought to enhance learning of complex cognitive tasks [3], lateralized song production may simplify the neural control required to generate and adaptively modify song production.

### Interhemispheric Switching during Song Production

Regardless of whether songbirds are phonating using one, or both sides of the syrinx, both hemispheres of the brain must be simultaneously active [45], [46]. This is due to the fact that during unilateral sound production, the nonphonating side of the syrinx typically closes to prevent airflow, although it may also remain open to silence the sound source [10], [24]. Therefore, bilateral hemispheric control is required to coordinate and regulate both the phonating and silent sides of the syrinx [10]. However, the bilateral neural activity during highly lateralized song production must be asymmetric because the motor control parameters are executing entirely different behavioral actions [46]. While acoustic phonology is controlled by one side of the song motor nuclei, the contralateral nuclei must prevent sound production.

The song of the Bengalese finch offers a unique opportunity to explore rapid interhemispheric switching of motor control during lateralized song production because the higher frequency syllables have a common pattern of right hemisphere control of the syllable onset, followed by left hemisphere control of the higher frequency note within the syllable (Figure 5). Although rapid interhemipsheric switching has been observed in zebra finches [13], this phenomenon may be far more pronounced and readily observed in Bengalese finches because of the distinct rapid switching between lower and higher frequency ranges to control the note structure within the syllable. Electrophysiological recordings of the neurons in the song motor nucleus RA (robust nucleus of the arcopallium) in Bengalese finches showed that pitch was the most pronounced correlation between neural activity and acoustic features of the song syllables [47]. These recordings were unilateral and primarily in the right hemisphere. It is possible that by comparing neural activity in the left RA nucleus to the right side, a stronger correlation with frequency would be observed. In addition, bilateral neural recordings would further our understanding of how the brain controls song because of the discrete patterns of syringeal control ranging from bilateral song production to rapid hemispheric switching in singing Bengalese finches.

### Evidence for Syringeal/Respiratory Compensation?

Air sac pressure results from the driving force of the respiratory muscles combined with the resistance to airflow through the syrinx. Eliminating or reducing gating may require respiratory compensation to adjust and maintain the similar respiratory patterns. Northern cardinals actively adjust respiratory effort and syringeal gating in response to a small perturbation in air sac pressure [48], [49]. Therefore, when syringeal gating of airflow is compromised, respiratory compensation may be required to maintain the respiratory patterns. However, it is possible that syringeal resistance contributes very little to the overall air pressure, and that the driving force from the respiratory muscles provides the majority of the control of the subsyringeal air pressure. Consistent with this possibility, peaks in the activity of expiratory muscles during zebra finch song usually coincide with increases in subsyringeal air pressure [18]. This illustrates the importance of respiratory muscles in controlling fine structure of air pressure and therefore also acoustic control of song. Future studies can explore whether or not Bengalese finches monitor and adjust respiratory pressure patterns, just as they rely on auditory feedback to shape ongoing vocal production.

### Summary

These data illustrate the importance of studying Bengalese finch song production to explore the functional significance of lateralized acoustic control. Experiments ranging from functional morphological studies of the syrinx, to the role of inter- and intra-sexual selection in shaping the acoustic control, lateralization of auditory processing, and whether lateralized song production increases a bird's ability to modify its song are promising areas of research. This line of research can also further our understanding of the functional significance of lateralized behaviors that are commonly observed across diverse groups of animals that perform complex, learned sensorimotor tasks, including speech and language. Furthermore, Bengalese finches provide an excellent model for exploring how different hemispheres interact and switch task control on very a short-time scale.

## Supporting Information

Audio S1 An example of song of a Bengalese finch recorded prior to left devocalization. The song syllables contain a mixture of higher and lower frequencies.
(WAV)

Audio S2 An example song recorded from the same bird in Audio S1 following left devocalization. Note the absence of higher frequencies in the song.
(WAV)

Audio S3 An example song recorded from a Bengalese finch prior to right syringeal devocalization.
(WAV)

Audio S4 An example song from the same bird from Audio S3 following right devocalization. Note the retention of higher frequency components in the bird's song.
(WAV)
